# Tissue Pharmacology of Da-Cheng-Qi Decoction in Experimental Acute Pancreatitis in Rats

**DOI:** 10.1155/2015/283175

**Published:** 2015-06-23

**Authors:** Xianlin Zhao, Yumei Zhang, Juan Li, Meihua Wan, Shifeng Zhu, Hui Guo, Jin Xiang, Edwin C. Thrower, Wenfu Tang

**Affiliations:** ^1^Department of Integrative Medicine, Sichuan Provincial Pancreatitis Center, West China Hospital, Sichuan University, Chengdu 610041, China; ^2^Department of Internal Medicine, Section of Digestive Diseases, Veterans Affairs Connecticut Healthcare of West Haven, Yale University School of Medicine, New Haven, CT 06520, USA

## Abstract

*Objectives*. The Chinese herbal medicine Da-Cheng-Qi Decoction (DCQD) can ameliorate the severity of acute pancreatitis (AP). However, the potential pharmacological mechanism remains unclear. This study explored the potential effective components and the pharmacokinetic characteristics of DCQD in target tissue in experimental acute pancreatitis in rats. *Methods*. Acute pancreatitis-like symptoms were first induced in rats and then they were given different doses of DCQD (6 g/kg, 12 g/kg, and 24 g/kg body weight) orally. Tissue drug concentration, tissue pathological score, and inflammatory mediators in pancreas, intestine, and lung tissues of rats were examined after 24 hours, respectively. *Results*. Major components of DCQD could be found in target tissues and their concentrations increased in conjunction with the intake dose of DCQD. The high-dose compounds showed maximal effect on altering levels of anti-inflammatory (interleukin-4 and interleukin-10) and proinflammatory markers (tumor necrosis factor *α* and interleukin-6) and ameliorating the pathological damage in target tissues (*P* < 0.05). *Conclusions*. DCQD could alleviate pancreatic, intestinal, and lung injury by altering levels of inflammatory cytokines in AP rats with tissue distribution of its components.

## 1. Introduction

Acute pancreatitis (AP) is an acute inflammation of the pancreas and surrounding tissue caused by pancreatic digestive enzymes. Although usually self-limiting, up to 20% of patients develop a severe form of disease, which can lead to a systemic inflammatory response syndrome (SIRS) and multiple organ dysfunction and failure [1]. It was reported that the overall mortality can range from 30 to 40% in the most severe cases [2, 3], and many are related with multiple organ dysfunction and failure [4–6]. Intestinal injury caused by AP is closely associated with poor prognosis in severe AP patients and is believed to be the primary cause of acute lung injury progression [7–10]. Recently, the adjuvant use of herbal medicine and electroacupuncture ameliorated lung and intestinal injury and led to a marked reduction in morbidity and mortality in China [11–14].

Da-Cheng-Qi Decoction (DCQD) is composed of Dahuang (*Radix et Rhizoma Rhei*), Houpu (*Magnolia officinalis REHD*.), Zhishi (*Fructus Aurantii Immaturus*), and Mangxiao (*Natrii Sulfas*) and has been widely used to treat AP for over 30 years in China [13, 15–17]. Recent studies have shown that DCQD can promote gastrointestinal motility, reduce acute lung injury, and inhibit cytokine activity and inflammatory responses in AP [15–18]. However, more detailed insights into the molecular mechanism of DCQD are hampered by difficulties in identifying which specific components of the complex mixture produce the observed therapeutic effects [19–21]. The pharmacological characteristics and bioactivities of DCQD inside the target tissue of AP rats are still not clear, even though there has been some progress in serum pharmacology [19, 22–24].

In the current study, we evaluated the tissue distribution of DCQD and related pharmacodynamic effects in pancreatic, lung, and intestinal tissue following administration of different doses of DCQD in experimental AP in rats. Such findings will prove helpful in further understanding the mechanism of DCQD in treatment of AP.

## 2. Materials and Methods

### 2.1. Animals

Thirty male clean-grade, healthy Sprague-Dawley rats (220 ± 15 g) were purchased from the Experimental Animal Center of West China Center of Medical Sciences of Sichuan University. All animal studies were fed, cared, and handled according to the Guide for the Care and Use of Laboratory Animals of Sichuan University and the Animal Ethics Committee Guidelines of the Animal Facility of the West China Hospital and China. They were acclimatized to the facilities for one week and fasted for 24 hours prior to the experiment.

### 2.2. Preparation of DCQD

The spray-dried drug powders of DCQD, Dahuang, Houpu, Zhishi, and Mangxiao, were purchased from Chengdu Green Herbal Pharmaceutical Co. Ltd. (Chengdu, China). Before being orally administered to rats, the spray-dried powder was mixed (12 : 24 : 12 : 9) by weight and reconstituted with sterile distilled water at different concentrations for the crude drug of 0.6 g/mL, 1.2 g/mL, and 2.4 g/mL.

### 2.3. Animal Models and Treatment with DCQD

The rats were randomly divided into normal group (NG), model group (MG), low-dose treatment group (LDG), median-dose treatment group (MDG), and high-dose treatment group (HDG). In this study, the rat model of AP was induced by retrograde perfusion into the biliopancreatic duct of 3.5% sodium taurocholate (Sigma, St. Louis, MO, USA) (1 mL/kg body weight) at a rate of 0.2 mL/min with a microinfusion pump [16, 25]. As a control, NG received the same procedure with saline (NaCl 0.9%) instead of sodium taurocholate. In DCQD-treated groups, the rats recovered from anesthesia and were administered intragastrically different concentrations of DCQD (10 mL/kg) in LDG (6 g/kg), MDG (12 g/kg), and HDG (24 g/kg) 2 h after the operation, respectively. In the NG and MG, rats were given an equal volume of saline with the same procedure.

### 2.4. Measurement of Histopathology of Pancreatic, Lung, and Intestinal Tissues

After 24 h following the procedure, rats were sacrificed and their pancreatic, lung, and intestinal tissues samples were collected for pathological examination and other analyses [11, 16]. Briefly, these collected tissues were fixed in 10% neutral formalin and embedded in paraffin, which were then cut into 5 *μ*m thick sections and stained with hematoxylin and eosin (HE) according to standard protocols. All the histopathology specimens were reviewed and scored in a blinded fashion by two independent pathologists using a scoring system for the extent and severity of tissue injury (0–4, edema, neutrophil infiltration, necrosis, and hemorrhage, resp.) as previously described [26–28]. The total histopathology score is the mean of the combined scores for each parameter from both investigators.

### 2.5. Measurement of Inflammatory Cytokine Levels in Tissues of Pancreas, Lung, and Intestine

The level of inflammatory cytokines, tumor necrosis factor *α* (TNF-*α*), interleukin-4 (IL-4), IL-10, and IL-6 in pancreas, and lung and intestine tissue samples were assessed using the Milliplex MAP Rat Cytokine/Chemokine magnetic bead immunoassay kit (Millipore Corporation, Billerica, MA) following the manufacturers' instructions [29, 30]. Briefly, diluted tissues homogenate samples and multiple premixed microbeads were added to a plate and the plate was incubated with agitation on a shaker. Then, the appropriate antibody detection mix was added into each well after washing. Each antibody was specific to a single cytokine. The plate was read on a MAGPIX Luminex xMAP instrument (Luminex Corp, Austin, TX) and analyzed with MILLIPLEX Analysis software version 3 (Millipore Corporation, Billerica, MA).

### 2.6. Measurement of Drug Concentrations of DCQD in Tissues of Pancreas, Lung, and Intestine

After 24 h following the procedure, pancreas, lung, and intestine tissues samples were collected and homogenized (10%) to measure the drug concentrations of DCQD. Similar to the analysis of serum pharmacokinetics of DCQD in our previous study, the concentrations of the 10 main components of DCQD (emodin, rhein, naringin, etc.), in pancreas, lung, and intestine tissue homogenates (10%), were measured by high performance liquid chromatography-mass spectrometry (HPLC-MS) as described [19, 23, 24].

Briefly, HPLC-MS system, consisting of a LC-10ADvp pump (Shimadzu), a SIL-HTc autosampler (Kyoto, Japan), and an API3000 triple-quadrupole LC-MS system (CA, USA), was controlled with Drug and Statistics analyst 1.4.2 software (Chinese Pharmacological Society, Beijing, China). Separation was performed on a C18 guard column (5 *μ*m, 4.0 mm × 2.0 mm, Phenomenex Inc., Torrance, CA, USA) and a YMC-Pack ODS-A C18 column (5 *μ*m, 150 mm × 4.6 mm, YMC, Kyoto, Japan). The mobile phase consisted of methanol-water (92 : 8, v/v) at a flow rate of 0.3 mL/min. The column was maintained at ambient temperature and the injection volume was 80 *μ*L. A mass spectrometer was operated using an electrospray source configured to the negative ion mode and quantification was performed by multiple reaction monitoring (MRM) [23].

Ten calibration standards were prepared by spiking 200 *μ*L of blank tissue with 100 *μ*L of each working solution to obtain tissue concentration of 400, 200, 100, 50, 25, 12.5, 6.25, and 3.13 ng/mL for emodin, 5000, 3750, 2500, 1250, 625, 312.5, 156.25, 78.13, 39.06, and 19.53 ng/mL for rhein, 160, 120, 80, 40, 20, 10, 5, 2.5, 1.25, and 0.63 ng/mL for rheochrysidin, and 800, 600, 400, 200, 100, 50, 25, 12.5, 6.25, and 3.13 ng/mL for aloe-emodin, chrysophanol, naringin, naringenin, hesperidin, magnolol, and honokiol. Quality control (QC) samples were prepared to obtain tissue concentrations of 3750, 625, 156.25, and 39.06 ng/mL for rhein, 100, 25, and 6.25 ng/mL for emodin, 600, 100, 25, and 6.25 ng/mL for aloe-emodin, chrysophanol, naringin, naringenin, hesperidin, magnolol, and honokiol, and 120, 20, 5, and 1.25 ng/mL for rheochrysidin. The spiked samples (standard and QC samples) were pretreated and detected in each analytical batch along with the unknown samples [23].

Data collection, peak integration, and calibration were all calculated with Analyst 1.4.2 software. Calibration curves were plotted according to the peak area ratio of analytes to internal standards (ibuprofen), and the linear regression between tissue concentration and peak area ratio was determined by 1/*x* ^2^. Concentrations of QC and unknown samples were measured by interpolation from the calibration curves [23, 24].

### 2.7. Statistical Analysis

All data were expressed as mean ± standard errors of mean (SEM). Statistical analysis was carried out using the PEMS3.1 statistical program. One-way repeated-measures ANOVA (followed by multiple pair-wise comparisons using Student-Neuman-Keuls procedure) was used for the analysis of differences among groups. For comparison, the level of statistical significance was set at *P* < 0.05 or less.

## 3. Results

### 3.1. DCQD Alleviated Pathological Damage in Pancreatic, Lung, and Intestinal Tissue

**Figure 1:**
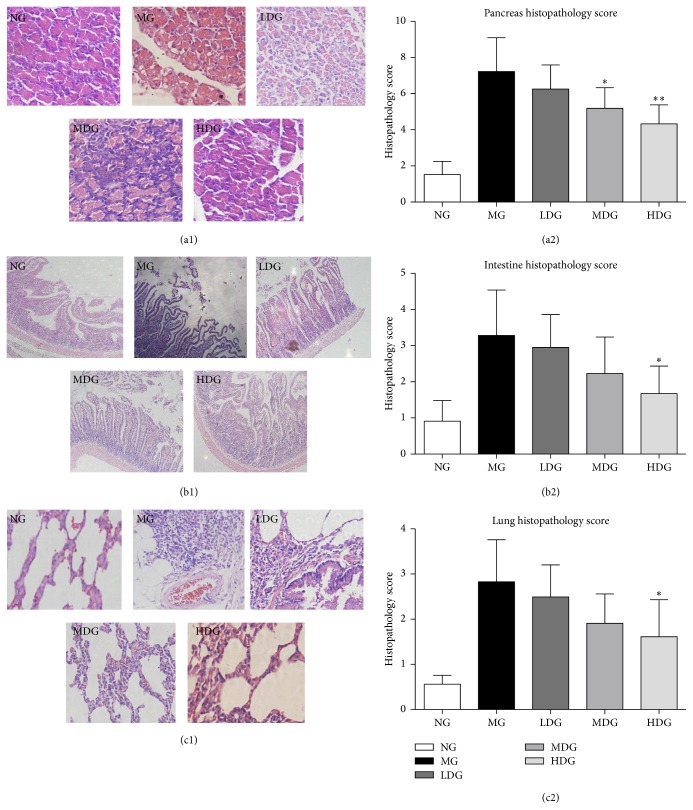
DCQD alleviated acute pancreatitis-associated tissue damage. Normal group = NG, model group = MG, low-dose group = LDG, medium-dose group = MDG, high-dose group = HDG. Rats (*n* = 6 per group) were given different dose of DCQD (6 g/kg in LDG, 12 g/kg in MDG, and 24 g/kg in HDG by body weight) 2 h after operation. After 24 h, the lung, intestine, and pancreas tissues were collected for pathological examination by hematoxylin and eosin (HE) staining. (a1) Pathological picture of pancreas (HE, ×400). (a2) Pathological scores of pancreas injury. (b1) Pathological picture of intestine (HE, ×100). (b2) Pathological scores of intestine injury. (c1) Pathological picture of lung (HE, ×400). (c2) Pathological scores of lung injury. The results are mean ± SE. ^*∗*^ *P* < 0.05 and ^*∗∗*^ *P* < 0.01 versus AP model group.

In NG, the pancreas exhibited no sign of edema and necrosis. However, the MG showed the features of AP characterized by expansion of interstitial edema, extensive infiltration of inflammatory cells, obvious pancreatic acinar cell vacuolization, necrosis, and hemorrhage. The rats treated with DCQD had a significant reduction of inflammatory cell infiltration, hemorrhaging, necrosis, and interstitial edema compared to MG, the greatest effect being seen in the HDG. DCQD reduced the standard pathological scores of the pancreas affected by experimental AP, and the scores of MDG and HDG were significantly lower than that in the MG at 24 hours (Figures 1(a1) and 1(a2)). Similar results could be found in intestinal and lung tissue of animals with experimental AP (Figures 1(b1), 1(b2), 1(c1), and 1(c2)).

### 3.2. DCQD Reduced Proinflammatory Cytokine Infiltration and Increased Anti-Inflammatory Cytokine Expression in Pancreatic, Lung, and Intestinal Tissue

**Figure 2:**
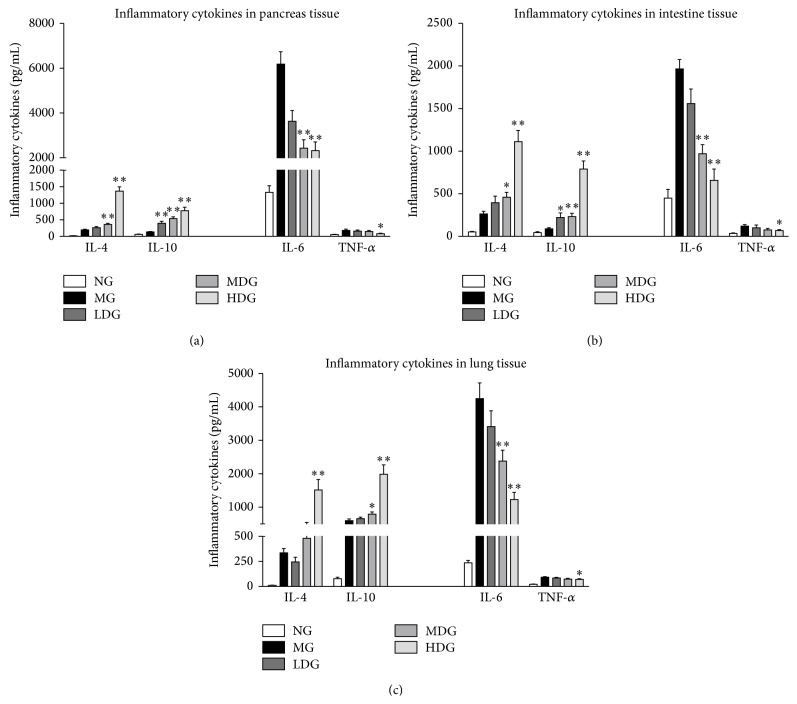
Effects of different dose of DCQD on the inflammatory cytokines in lung, intestine, and pancreas tissues. Normal group = NG, model group = MG, low-dose group = LDG, medium-dose group = MDG, high-dose group = HDG. Rats (*n* = 6 per group) were given different dose of DCQD (6 g/kg in LDG, 12 g/kg in MDG, and 24 g/kg in HDG by body weight) 2 h after operation. After 24 h, the lung, intestine, and pancreas tissues were collected for examination of proinflammatory cytokines (IL-6 and TNF-*α*) and anti-inflammatory cytokines (IL-4 and IL-10). (a) Inflammatory cytokines in pancreas tissues. (b) Inflammatory cytokines in intestine tissues. (c) Inflammatory cytokines in lung tissues. The results are mean ± SE. ^*∗*^ *P* < 0.05 and ^*∗∗*^ *P* < 0.01 versus AP model group.

In the AP model group, there was a significant increase of inflammatory cytokine infiltration of pancreatic tissue at 24 h after sodium taurocholate stimulation compared with the sham operation group, especially the proinflammatory cytokine IL-6. In the DCQD-treated group, proinflammatory cytokine (IL-6 and TNF-*α*) infiltration was significantly lower, and the anti-inflammatory cytokine levels (IL-4 and IL-10) were significantly higher than that in the AP model group (*P* < 0.05) (Figure 2(a)). The greatest effect was seen in the HDG (*P* < 0.01). Similar results were found in intestine and lung tissues of experimental AP (Figures 2(b) and 2(c)).

### 3.3. Major Components of DCQD Become Distributed in Pancreatic, Lung, and Intestinal Tissue

**Figure 3:**
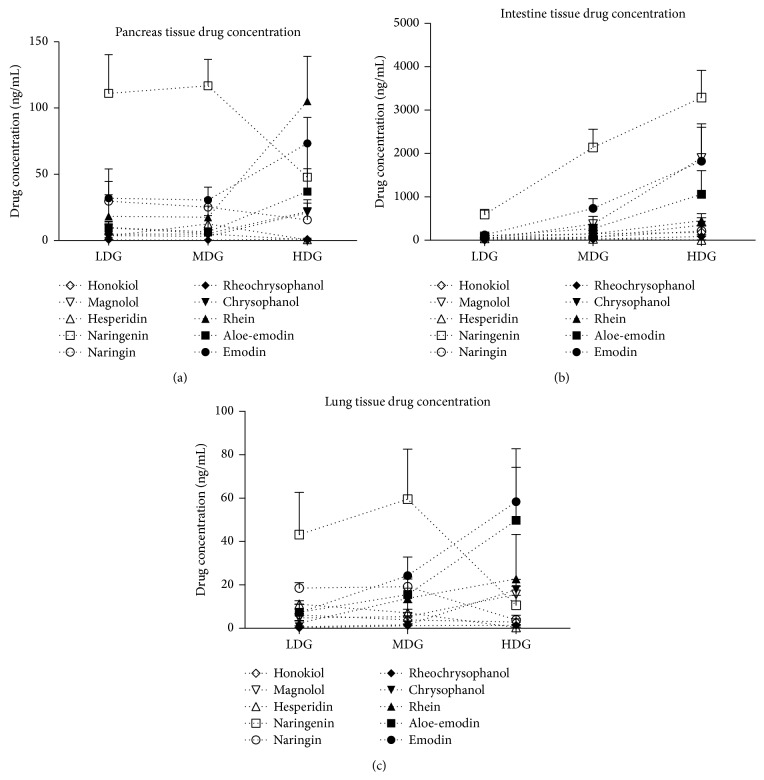
Distribution of different dose of DCQD in lung, intestine, and pancreas tissues. Normal group = NG, model group = MG, low-dose group = LDG, medium-dose group = MDG, high-dose group = HDG. Rats (*n* = 6 per group) were given different dose of DCQD (6 g/kg in LDG, 12 g/kg in MDG, and 24 g/kg in HDG by body weight) 2 h after operation. After 24 h, the lung, intestine, and pancreas tissues were collected for examination of drug concentration of DCQD. (a) Drug concentration in pancreas tissues. (b) Drug concentration in intestine tissues. (c) Drug concentration in lung tissues. The results are mean ± SE.

Concentrations of major components of DCQD in pancreatic, lung, and intestinal tissue of rats treated with DCQD were measured using a sensitive HPLC-TMS method. The result showed that the five major components of DCQD that were most abundant in the pancreatic tissue of rats after oral administration are naringenin, naringin, rhein, aloe-emodin, and emodin. The concentration of these components increased concurrently with the oral dose of DCQD other than naringenin (Figure 3(a)). Similar results could be found in intestinal and lung tissues (Figures 3(b) and 3(c)). The highest tissue component level of DCQG was rhein in pancreas, naringenin in intestine, and emodin in lung when given high-dose DCQD, respectively (Figure 3). It also was found that the concentration of the major components of DQCD in intestinal tissue was higher than that in pancreas and lung tissue.

## 4. Discussion

The biological effects of specific DCQD components and their tissue distribution have been a subject for much discussion. Previous studies have determined 4–10 major constituents of DCQD in dog and rat serum by HPLC-TMS method [19, 22, 23, 31], but the targeting of specific tissues, such as pancreas, lung, and intestine, by these components remain unclear. This study showed that the most abundant components of DCQD in target tissue of rats had a similar distribution with that seen in serum after oral administration [19, 31]. However, the concentration of the major components of DCQD, such as naringenin, naringin, rhein, aloe-emodin, and emodin, in intestinal tissue was higher than that in pancreas and lung tissue. These findings indicate that major components of DCQD may be absorbed from the intestine and can end up in target tissue via the blood transport system. Our finding is consistent with the hypothesis of tissue pharmacology [32]. That is to say that the specific tissue pharmacology of components of Chinese medicine differs, depending on the target tissue; for example, the maximal levels of rhein were found in pancreas, emodin in lung, and naringenin in intestine, respectively. The blood-tissue barriers in different tissue may explain these phenomena. At present, most studies focus on the blood-brain barrier and gut barrier, whereas research regarding blood-pancreas barrier and blood-lung barrier is rare. The only study to touch upon this was from Burns et al. [33] who reported a role for the blood-pancreatic barrier in antibiotic excretion in chronic pancreatitis decades ago. Future research should focus on the blood-pancreatic barrier as this may play an important role in treatment of AP, given that the pancreas is the origin of inflammatory cytokines [6, 34].

AP is a common and potentially lethal acute inflammatory disease, which is thought to be mediated by a variety of pro- and anti-inflammatory mediators released from the pancreas and various other sources during the course of the disease [34, 35]. Local recruitment and activation of inflammatory cells in injured pancreas may lead to the production of proinflammatory cytokines, such as IL-6 and TNF-*α*, as well as anti-inflammatory IL-4 and IL-10. The imbalance of proinflammatory and anti-inflammatory cytokines could lead to the inflammatory cascade and injure intestine and lung tissue, which aggravate the progression of AP [10, 36]. In this study, we examined the value of IL-4, IL-6, IL-10, and TNF-*α* as predictors of inflammation in AP. Our result showed that the level of IL-6 was higher than other inflammatory cytokines and the level of TNF-*α* was lowest in pancreas, lung, and intestinal tissues at 24 h. The role of proinflammatory IL-6 in predicting severity of acute pancreatitis seems much more valuable than TNF-*α*. DCQD treatment could increase the expression of anti-inflammatory cytokines (IL-4 and IL-10) and inhibit the expression of proinflammatory cytokines (TNF-*α* and IL-6) to ameliorate the histopathological damage of acute pancreatitis, in which higher dose DCQD seems to have the better effect. These data support a DCQD-regulated systemic proinflammatory media/anti-inflammatory media balance in rats. Similar results can be found in other studies. Our previous studies found that rats treated with DCQD had lower mean pathological lung lesion scores, lower level of IL-6 mRNA, and higher expression of IL-10 mRNA than those in AP rats [18]. Huang et al. [12] reported that revised DCQD may inhibit IL-6 induction and increase IL-10 concentration and HSP70 expression, effectively reducing lung injury. It is important to note that some research studies reported that certain monomer components of DCQD can also exert anti-inflammatory actions. For example, Gao et al. [37] reported that rhein could inhibit nuclear factor-*κ*B (NF-*κ*B) activation and sequentially suppresses its downstream inducible nitric oxide synthase, IL-6, and TNF-*α* by inhibiting IKK*β* in LPS-activated macrophages. Han et al. [38] reported that emodin treatment could attenuate IL-1*β* secretion via the inhibition of NOD-like receptor family, pyrin domain containing 3 (NLRP3) inflammasome activation in LPS-induced endotoxin mouse models. In view of the close relationship between DCQD and inflammatory cytokines, future research should focus on the link between quantified molecules of DCQD and their pharmacological effects with respect to their target tissue.

In summary, we confirmed that major components of DCQD could be absorbed into the target tissue of AP and ameliorate the histopathological damage by increasing the expression of the anti-inflammatory cytokines and inhibiting the expression of proinflammatory cytokines, which may be associated with the intake dose of DCQD. Further studies should investigate these ingredients in greater detail to take into account the specific molecular mechanisms in treatment of AP and help optimize herbal formulations as a therapy.
